# Efficacy of non-invasive brain stimulation for disorders of consciousness: a systematic review and meta-analysis

**DOI:** 10.3389/fnins.2023.1219043

**Published:** 2023-07-11

**Authors:** Linghui Dong, Hui Li, Hui Dang, Xiaonian Zhang, Shouwei Yue, Hao Zhang

**Affiliations:** ^1^Shandong University, Jinan, Shandong, China; ^2^China Rehabilitation Research Center, Beijing, China; ^3^University of Health and Rehabilitation Sciences, Qingdao, Shandong, China

**Keywords:** non-invasive brain stimulation (NIBS), transcranial direct current stimulation (tDCS), repetitive transcranial magnetic stimulation (rTMS), transcranial random noise stimulation (tRNS), disorders of consciousness (DOC), meta-analysis

## Abstract

**Objective:**

The aim of this study is to evaluate the efficacy of non-invasive brain stimulation (NIBS) in patients with disorders of consciousness (DoC) and compare differences in efficacy between different stimulation modalities.

**Methods:**

We searched the PubMed, Cochrane Library, Web of Science, and EMBASE databases for all studies published in English from inception to April 2023. Literature screening and quality assessment were performed independently by two investigators. Weighted mean differences (WMDs) and 95% confidence intervals (CIs) were used to evaluate the therapeutic effects of NIBS. The Cochrane Q test and I^2^ statistic were used to evaluate heterogeneity between studies. Subgroup analysis was performed to identify the source of heterogeneity, and differences in efficacy between different stimulation modalities were compared by Bayesian analysis.

**Results:**

A total of 17 studies with 377 DoC patients were included. NIBS significantly improved the state of consciousness in DoC patients when compared to sham stimulation (WMD: 0.81; 95% CI: 0.46, 1.17; I^2^ = 78.2%, *p* = 0.000). When divided into subgroups according to stimulation modalities, the heterogeneity of each subgroup was significantly lower than before (I^2^: 0.00–30.4%, *p* >0.05); different stimulation modalities may be the main source of such heterogeneity. Bayesian analysis, based on different stimulation modalities, indicated that a patient’s state of consciousness improved most significantly after repetitive transcranial magnetic stimulation (rTMS) of the left dorsolateral prefrontal cortex (DLPFC). Diagnosis-based subgroup analysis showed that NIBS significantly improved the state of consciousness in patients with a minimal consciousness state (WMD: 1.11; 95% CI: 0.37, 1.86) but not in patients with unresponsive wakefulness syndrome or a vegetative state (WMD: 0.31; 95% CI: −0.09, 0.71). Subgroup analysis based on observation time showed that single treatment did not improve the state of consciousness in DoC patients (WMD: 0.28; 95% CI: −0.27, 0.82) while multiple treatments could (WMD: 1.05; 95% CI: 0.49, 1.61). Furthermore, NIBS had long-term effects on DoC patients (WMD: 0.79; 95% CI: 0.08–1.49).

**Conclusion:**

Available evidence suggests that the use of NIBS on patients with DoC is more effective than sham stimulation, and that rTMS of the left DLPFC may be the most prominent stimulation modality.

## 1. Introduction

With the development of emergency and critical care medicine, the mortality rate of neurocritically ill patients has significantly decreased, and the incidence of disorders of consciousness (DoC) among survivors has dramatically increased (Schnakers, 2020). DoC refers to the loss of consciousness caused by various severe brain injuries; the mechanisms involved may be related to severe structural damage in the brain, thus causing widespread regression of excitatory synaptic activity and changes in the concentration of key biochemical substances such as local transmitters (Clauss, 2014). If this condition extends for more than 28 days, it is known as prolonged disorders of consciousness (pDoC) (Pundole et al., 2021). Currently, the rehabilitation of DoC patients remains a worldwide conundrum and involves a long and difficult rehabilitation cycle. Patients require long-term personal care, thus creating a heavy burden on their families and society. Consequently, it is vital that we investigate the specific mechanisms underlying the recovery of consciousness in DoC patients and identify effective interventions.

DoC can be divided into different phases: (1) coma, a deep state of unconsciousness without any signs of wakefulness and only reflex behavior, (2) unresponsive wakefulness syndrome (UWS), previously known as a vegetal state (*VS*) in which an individual awakens from coma, thus indicating the presence of a sleep/wakefulness cycle and arousal, but not consciousness, and (3) a minimally consciousness state (MCS) in which patients exhibit functional communication or the use of functional objectives (Eapen et al., 2017). At present, the assessment of chronic DoCs is mainly based on the revised coma recovery scale (CRS-R), combined with neuroimaging, electrophysiology, and other methods that can accurately respond to a patient’s true level of consciousness. Compared to *VS*/UWS patients, MCS patients have relatively preserved brain function and can exhibit discrete and fluctuating well-defined signs of consciousness with high plasticity and recovery potential (Thibaut et al., 2019).

The mechanisms underlying the occurrence and recovery of DoC remain unclear, and findings based on neuroimaging techniques such as functional magnetic resonance imaging (fMRI) suggest that the maintenance of consciousness may be related to the functional connectivity (the consistency of neural activity across time series) within various brain regions and brain networks. The default mode network (DMN), which is typically activated to a high state when individuals are awake at rest and not focused on the outside world, is involved in memory consolidation and the large-scale integration of consciousness related signals (Mäki-Marttunen et al., 2016). Functional connectivity within or with other networks within the DMN is of significant value for predicting consciousness awareness, with significant correlations between the strength of functional connectivity and the level of consciousness (Wu et al., 2015; Threlkeld et al., 2018). In addition to the DMN, extensive and severe impairment of the frontoparietal network (FPN) may also contribute to the occurrence of DoC (Cauda et al., 2009). FPN is involved in constituting a key neural basis for the spatial consciousness regulation of the brain’s overall network and is negatively correlated with the DMN, mainly comprising the executive control network (ECN) and dorsal attachment network (Dan) (Kelly et al., 2008; Stawarczyk et al., 2018; Mallas et al., 2021). In addition, the thalamus also plays an important role in maintaining consciousness, and its connectivity with the DMN and FPN is closely related to the maintenance of consciousness; this improvement in connectivity could facilitate the recovery of consciousness in patients with DoC (Crone et al., 2014; He et al., 2015).

Current treatments for DoC mainly include pharmacotherapy (e.g., amantadine) and rehabilitation (e.g., exercise therapy, multisensory stimulation, and hyperbaric oxygen), although the therapeutic effects of these modalities are not ideal (Thibaut et al., 2019). Of these, amantadine, a non-competitive glutamate receptor antagonist, is the only treatment that is currently recommended for DoC by the American Academy of Neurology (Thibaut et al., 2019) and is able to accelerate the release of dopamine from nerve terminals, reduce dopamine uptake, and increase the neuronal levels of dopamine (Ma and Zafonte, 2020).

In recent years, given the paucity of treatments available for patients with DoC, researchers have begun to actively explore neuroplasticity based regulatory modalities to awaken patients with DoC to a ‘sleepy brain’ state. Of these, non-invasive brain stimulation (NIBS) has been applied in the treatment of DoC patients due to the fact that this method is non-invasive, painless, safe, and can directly modulate cortical excitability. The use of NIBS in patients with DoC includes transcranial direct current stimulation (tDCS) (Thibaut et al., 2014; Estraneo et al., 2017; Huang et al., 2017; Thibaut et al., 2017; Zhang et al., 2017; Martens et al., 2018, 2019; Wu et al., 2019; Carrière et al., 2020; Martens et al., 2020), repetitive transcranial magnetic stimulation (rTMS) (Cincotta et al., 2015; Naro et al., 2015; He et al., 2018; Liu et al., 2018; He et al., 2021; Fan et al., 2022), and transcranial random noise stimulation (tRNS) (Mancuso et al., 2017). More commonly applied for patients with DoC, tDCS often involves anode placement over the left dorsolateral prefrontal cortex (DLPFC), a technique that affects cortical excitability by inducing a weak current (usually 1–2 MA) between two electrodes (anode and cathode) placed over the scalp (Lefaucheur et al., 2017). RTMS is the modulation of excitability in targeted brain regions by means of a rapidly changing magnetic field; this strategy forms microcurrents directly across the cerebral cortex *via* extracerebral tissues (scalp, bone, meninges) (Lefaucheur et al., 2020). High frequency tRNS is a recently developed transcranial stimulation modality capable of exerting long-lasting effects on cortical excitability when acting on the cerebral cortex based on the electrical oscillatory spectrum in the form of white noise (Snowball et al., 2013; Mancuso et al., 2017).

Although the use of NIBS in patients with DoC has achieved limited results, further meta-analysis is needed for more accurate evaluation of its efficacy due to the lack of high-quality RCTs with large sample sizes. In addition, because the effect of NIBS is influenced by a number of factors, including patient-specific factors, stimulation modality and treatment cycle, this study conducted subgroup analysis of NIBS based on a number of different factors. We also performed Bayesian analysis on stimulation modality to investigate the effect of each factor on the efficacy of NIBS and to identify the most effective stimulation modality so as to provide a reference for the clinical application of NIBS.

## 2. Methods

### 2.1. Search strategy

This meta-analysis was performed according to the preferred reporting items for systematic reviews and meta-analyses (PRISMA) (Moher et al., 2009) and was registered with Prospero (CRD42022361237). We searched the PubMed, Cochrane Library, Web of Science, and EMBASE databases for all studies published from inception to April 2023. We limited our search to studies published in English. The keywords for identifying NIBS were as follows: non-invasive brain stimulation, transcranial direct current stimulation, repetitive transcranial magnetic stimulation, theta burst stimulation, transcranial random noise stimulation and transcranial ultrasound stimulation. The key words used to identify DoC were as follows: disorders of consciousness, prolonged disorders of consciousness, coma, unresponsive wakefulness syndrome, vegetative state, and minimal consciousness state. In addition, reference lists from relevant studies were manually screened to identify other articles that may have been omitted. Two researchers independently completed the search and read and identified all titles to exclude irrelevant papers. Any contradictions between the two researchers were resolved by discussion.

### 2.2. Study selection

#### 2.2.1. Inclusion criteria

The Population-Intervention-Comparison-Outcomes-Study design (PICOS) framework (Schardt et al., 2007) was used to develop the inclusion criteria: (P) Population, studies enrolling adult DoC patients; (I) Intervention, studies involving cortex-targeted NIBS; (C) Comparison, studies with sham stimulation as a control; (O) Outcomes, studies assessing intervention efficacy with the CRS-R, and (S) Study design, randomized controlled trials of parallel design and crossover design, for comprehensive considerations.

#### 2.2.2. Exclusion criteria

We excluded non-randomized controlled studies; studies in which sham stimulation was not performed in controls; reviews, case reports, and meeting abstracts; studies which featured an insufficient data set, and studies that were not published in English.

### 2.3. Quality assessment

The quality of the included studies was assessed according to The Cochrane Collaboration’s tool: random sequence generation, allocation consensus, blinding of participants and personnel, blinding of outcome assessment, incomplete outcome data, selective reporting, and other bias (Higgins et al., 2011). Two investigators independently completed the assessment of study quality, and discrepancies in the assessment were resolved by discussion until a consensus was reached.

### 2.4. Data extraction

The following data were extracted from the full texts of the included studies: the first author’s name, year of publication, study design, sample size, demographic information relating to the patients (age, sex, patient diagnosis, ethics, time after injury), NIBS protocol (type of stimulation, stimulation target, stimulation intensity, single dose, treatment cycle), efficacy indicators (CRS-R score before and after intervention), and advantage effect.

### 2.5. Statistical analysis

The effect of NIBS on consciousness functioning in DoC patients was defined as the mean difference in the change from baseline CRS-R score between the experimental and control groups. As the included studies used uniform outcome measures, the results were expressed as weighted mean differences (WMDs) and 95% confidence intervals (CIs). *p*-values <0.05 were considered statistically significant. When studies did not present changes in CRS-R scores, the following formula was used: mean change = mean final mean baseline. For studies where raw data were shown as mean ± SES, the SD was calculated as: SES = SD/ $\sqrt{n}$ (n indicates the number of participants). If results were reported as quartiles, then we estimated means (Luo et al., 2018) and SDS (Shi et al., 2020) using the following formula in which, *a* = the minimum value, *q*_1_ = the first quartile, *m* = the median, *q*_3_ = the third quartile, *b* = the maximum value:


$$\bar{X}\approx \frac{a+2q_{1}+2m+2q_{3}+b}{8}$$

$$\begin{array}{l} S\approx [\frac{\left(a^{2}+2q_{1}^{2}+2m^{2}+2q_{3}^{2}+b^{2}\right)}{16}+\frac{\left(aq_{1}+q_{1}m+mq_{3}+q_{3}b\right)}{8}\\ \;\;\;\;\;\;\;\;\;\;\;{-\frac{{\left(a+2q_{1}+2m+2q_{3}+b\right)}^{2}}{16}]}^{1/2} \end{array}$$

We used the Q test and I^2^ statistic to evaluate the heterogeneity of the included literature. A fixed effects model was used when I^2^ values were  < 50% and random models were used to summarize effect sizes when I^2^ values were  ≥ 50%. The Galbraith plot method was used to identify outliers with heterogeneity in meta-analysis. Galbraith plot provides a graphical display that intuitively discovers heterogeneous studies. For each study, divide WMD by its standard error as the vertical axis and the reciprocal of standard error as the horizontal axis. There is significant heterogeneity in studies located outside the confidence interval. If outliers were present, the study was re-examined and heterogeneity changes were observed after exclusion, further determining whether the study was excluded.

To further investigate factors that may mediate the effects of NIBS on the state of consciousness, the following three subgroup analyses were implemented: stimulation modality (left DLPFC tDCS vs. left DLPFC rTMS vs. left M1 rTMS; other stimulation modalities were not analyzed as a subgroup alone due to only one study being involved): diagnosis (*VS*/UWS vs. MCS), observation time (single treatment vs. multiple treatments vs. long term efficacy). Bayesian analysis was based on different stimulation modalities.

Stata 12.0 software (StataCorp LLC, United States) was used for meta-analysis and subgroup analysis, and Addis 1.16.6 software was used for Bayesian analysis. Funnel plots were constructed, and Egger’s test was performed to assess publication bias. Where publication bias was present, clipping was applied for correction. Sensitivity analysis was performed by applying the single article exclusion method.

## 3. Results

### 3.1. Search results

**Figure 1:**
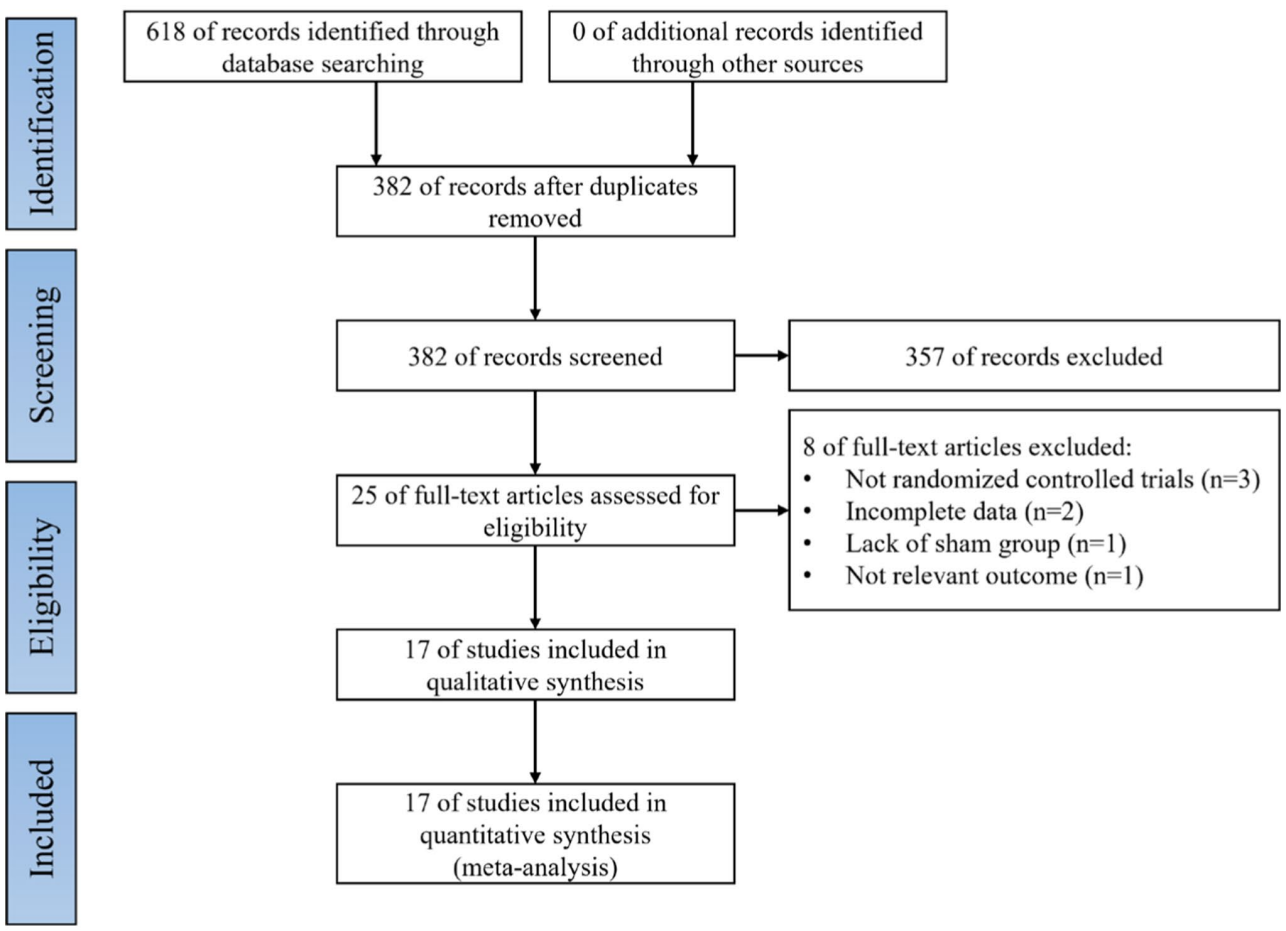
Flow diagram.

Literature searches were performed for all selected databases and yielded a total of 618 articles; 236 duplicates were excluded by Endnote 20 software (Clarivate PLC, United States). The remaining 382 articles and abstracts were scanned, and 357 entries were excluded because the subject matter did not meet or did not obviously meet the inclusion criteria. Full text evaluation was performed for the remaining 25 articles; three were excluded because they were non-RCTs, two were excluded because of incomplete data, one was excluded because of controls that did not involve sham stimulation, and one was excluded because it reported irrelevant results. Finally, 17 studies were included in the final meta-analysis (Thibaut et al., 2014; Cincotta et al., 2015; Naro et al., 2015; Estraneo et al., 2017; Huang et al., 2017; Mancuso et al., 2017; Thibaut et al., 2017; Zhang et al., 2017; He et al., 2018; Liu et al., 2018; Martens et al., 2018, 2019; Wu et al., 2019; Carrière et al., 2020; Martens et al., 2020; He et al., 2021; Fan et al., 2022). A flow diagram of the literature search is shown in Figure 1.

### 3.2. Characteristics of the studies

The 17 included studies involved a total of 377 DoC patients; the basic characteristics of the studies and patients are shown in Table 1. Sixteen studies featured one intervention group (Thibaut et al., 2014; Cincotta et al., 2015; Naro et al., 2015; Estraneo et al., 2017; Huang et al., 2017; Mancuso et al., 2017; Thibaut et al., 2017; Zhang et al., 2017; He et al., 2018; Liu et al., 2018; Martens et al., 2018, 2019; Carrière et al., 2020; Martens et al., 2020; He et al., 2021; Fan et al., 2022) and one study featured two intervention groups (Wu et al., 2019). Six studies employed left DLPFC tDCS (Thibaut et al., 2014; Estraneo et al., 2017; Thibaut et al., 2017; Zhang et al., 2017; Martens et al., 2018; Carrière et al., 2020), three studies employed the left M1 rTMS (Cincotta et al., 2015; He et al., 2018; Liu et al., 2018), two studies employed left DLPFC rTMS (He et al., 2021; Fan et al., 2022), one study employed left or right DLPFC tDCS (Wu et al., 2019), one study employed left or right M1 tDCS (Martens et al., 2019), one study employed right DLPFC rTMS (Naro et al., 2015), one study employed bilateral frontoparietal areas tDCS (Martens et al., 2020), one study employed left posterior parietal cortex tDCS (Huang et al., 2017) and one study employed biliary DLPFC tRNS (Mancuso et al., 2017).

**Table 1:** The basic characteristics of the studies and patients.

Authors, years	Study design	Participants (*N*; Age: m ± SD years; Gender; Patient diagnosis; Etiology)	Time after injury	NIBS protocol (experimental vs. control)	Stimulation target	Adverse effect
Carrière et al. (2020)	Crossover study	*N* = 10, Age = 47 ± 15.13 Males = 7, Females = 3 MCS = 10 TBI = 3, Vascular = 4, Cardiogenic = 3, Meningitis = 1	8.2 ± 6.96 months	20 min 2 mA tDCS 20 min sham tDCS 1 session, 48 h washout	Left DLPFC	None
Cincotta et al. (2015)	Crossover study	*N* = 11, Age = 59.64 ± 13.83 Males = 7, Females = 4 *VS*/UWS = 11 TBI = 2, Anoxia = 9	35.36 ± 25.84 months	1,000 pulses, 20 Hz, 90% rMT rTMS 1,000 pulses, sham rTMS 5 sessions, 1 month washout	Left M1	None
Estraneo et al. (2017)	Crossover study	*N* = 13, Age = 54.54 ± 21.64 Males = 7, Females = 6 MCS = 6, *VS*/UWS = 7 TBI = 1, Vascular = 6, Anoxic = 6	20 ± 20.54 months	20 min 2 mA tDCS 20 min sham tDCS 5 sessions, 1 week washout	Left DLPFC	None
Fan et al. (2022)	Parallel study	*N* = 40, Age = 48.9 ± 13.67 Males = 25, Females = 15 *VS*/UWS = 40 TBI = 15, Vascular = 25	1.69 ± 0.92 months	2000 pulses, 20 Hz, 100% rMT rTMS 2000 pulses, sham rTMS 20 sessions	Left DLPFC	None
He et al. (2018)	Crossover study	*N* = 6, Age = 39.5 ± 15.68 Males = 4, Females = 2 MCS = 3, *VS*/UWS = 3 TBI = 4, Vascular = 2	8.17 ± 10.21 months	1,000 pulses, 20 Hz, 100% rMT rTMS 1,000 pulses, sham rTMS 5 sessions, 1 week washout	Left M1	None
He et al. (2021)	Parallel study	*N* = 50, Age = 52.05 ± 2.51 Females = 50 MCS = 14, *VS*/UWS = 36 TBI = 14, Vascular = 34, Anoxic = 2	2.87 ± 0.57 months	1,000 pulses, 10 Hz, 100% rMT rTMS 1,000 pulses, sham rTMS 10 sessions	Left DLPFC	None
Huang et al. (2017)	Crossover study	*N* = 33, Age = 57 ± 11 Males = 20, Females = 13 MCS = 33 TBI = 20, non-TBI = 13	6 ± 5 months	20 min 2 mA tDCS 20 min sham tDCS 5 sessions, 5 days washout	Left Posterior parietal cortex	None
Liu et al. (2018)	Crossover study	*N* = 7, Age = 48 ± 16.57 Males = 6, Females = 1 MCS = 5, *VS*/UWS = 2 TBI = 5, Vascular = 1, Anoxic = 1	3.14 ± 1.86 months	1,000 pulses, 20 Hz, 100% rMT rTMS 1,000 pulses, sham rTMS 5 sessions, 1 week washout	Left M1	None
Mancuso et al. (2017)	Parallel study	*N* = 9, Age = 71.67 ± 10 Males = 3, Females = 6 *VS*/UWS = 9 TBI = 1, Vascular = 5, Anoxic = 3	1.51 ± 1.06 months	20 min, 5 mA, 101–640 Hz tRNS 20 min sham tRNS 5 sessions	Bilateral DLPFC	None
Martens et al. (2018)*	Crossover study	*N* = 27, Age = 42 ± 14.47 Males = 19, Females = 8 MCS = 27 TBI = 12, Vascular = 5, Cardiogeni = 9, Anoxic = 1	96.89 ± 31.79 months	20 min 2 mA tDCS 20 min sham tDCS 20 sessions, 8 weeks washout	Left DLPFC	Skin redness in 12 patients, Sleppiness in 5 patients, Epileptic seizure in 1 patient
Martens et al. (2019)*	Crossover study	*N* = 10, Age = 49.1 ± 22.64 Males = 8, Females = 2 MCS = 6, *VS*/UWS = 4 TBI = 5, non-TBI = 5	7.25 ± 13.42 months	20 min 2 mA tDCS 20 min sham tDCS 1 session, 24 h washout	Left or right M1	None
Martens et al. (2020)	Crossover study	*N* = 46, Age = 46.33 ± 15.02 Males = 27, Females = 19 MCS = 29, *VS*/UWS = 17 TBI = 22, non-TBI = 24	36.94 ± 65.19 months	20 min 1 mA tDCS 20 min sham tDCS 1 session, 48 h washout	Bilateral frontoparietal	None
Naro et al. (2015)*	Crossover study	*N* = 3, Age = 36.67 ± 4.16 Males = 1, Females = 2 *VS*/UWS = 3 Anoxic = 3	10.67 ± 5.86 months	1,000 pulses, 10 Hz, 90% rMT rTMS 1,000 pulses, sham rTMS 1 session, 1 week washout	Right DLPFC	None
Thibaut et al. (2014)	Crossover study	*N* = 55, Age = 43.09 ± 17.92 Males = 40, Females = 15 MCS = 30, *VS*/UWS = 25 TBI = 25, Vascular = 15, Anoxic = 13, TBI- anoxic = 2	33.29 ± 56.5 months	20 min 2 mA tDCS 20 min sham tDCS 1 session, 48 h washout	Left DLPFC	None
Thibaut et al. (2017)	Crossover stud	*N* = 16, Age = 43.31 ± 15.65 Males = 9, Females = 7 MCS = 16 TBI = 11, Vascular = 2, Cardiogeni = 3	78.85 ± 100.83 months	20 min 2 mA tDCS 20 min sham tDCS 5 sessions, 1 week washout	Left DLPFC	None
Wu et al. (2019)*	Parallel study	*N* = 15, Age = 47.87 ± 17.83 Males = 9, Females = 6 MCS = 7, *VS*/UWS = 8 TBI = 5, Vascular = 7, Anoxic = 3	152.8 ± 153.3 months	20 min 2 mA tDCS 20 min sham tDCS 10 sessions	Left or right DLPFC	None
Zhang et al. (2017)	Parallel stud	*N* = 26, Age = 52.69 ± 20.17 Males = 15, Females = 11 MCS = 15, *VS*/UWS = 11 TBI = 12, Vascular = 9, Anoxic = 5	5.28 ± 4.14 months	20 min 2 mA tDCS 20 min sham tDCS 20 sessions	Left DLPFC	None

Martens et al. (2018)* reported only one patient with seizures during the intervention; however, this patient received sham stimulation. Martens et al. (2019)* only featured one intervention group; the tDCS anode was placed in the M1 region of the cerebral hemisphere on the most damaged side. Naro et al. (2015)* which was a randomized controlled trial with a crossover design, only performed active and sham stimulation in three patients; data from seven patients who did not undergo sham stimulation were excluded. Wu et al. (2019)* described two intervention groups that received left DLPFC tDCS or right DLPFC tDCS.

### 3.3. Quality assessment

**Figure 2:**
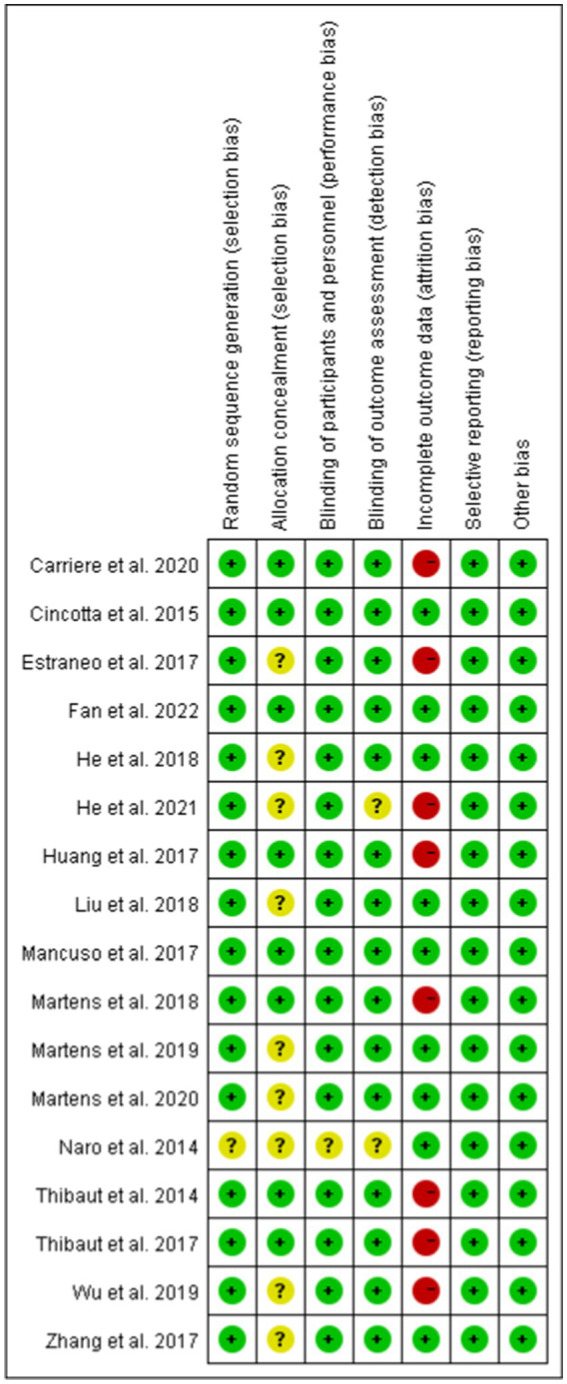
Risk of bias assessments.

We used the Cochrane scoring system to assess the quality of the included studies. The risk of bias was assessed by the following seven criteria: random sequence generation, allocation consensus, blinding of participants and personnel, blinding of outcome assessment, incomplete outcome data, selective reporting, other bias. Studies were classified as low risk of bias, high risk of bias, and unclear risk. Risk of bias assessments are detailed in Figure 2.

### 3.4. The effects of NIBS on DoC

**Figure 3:**
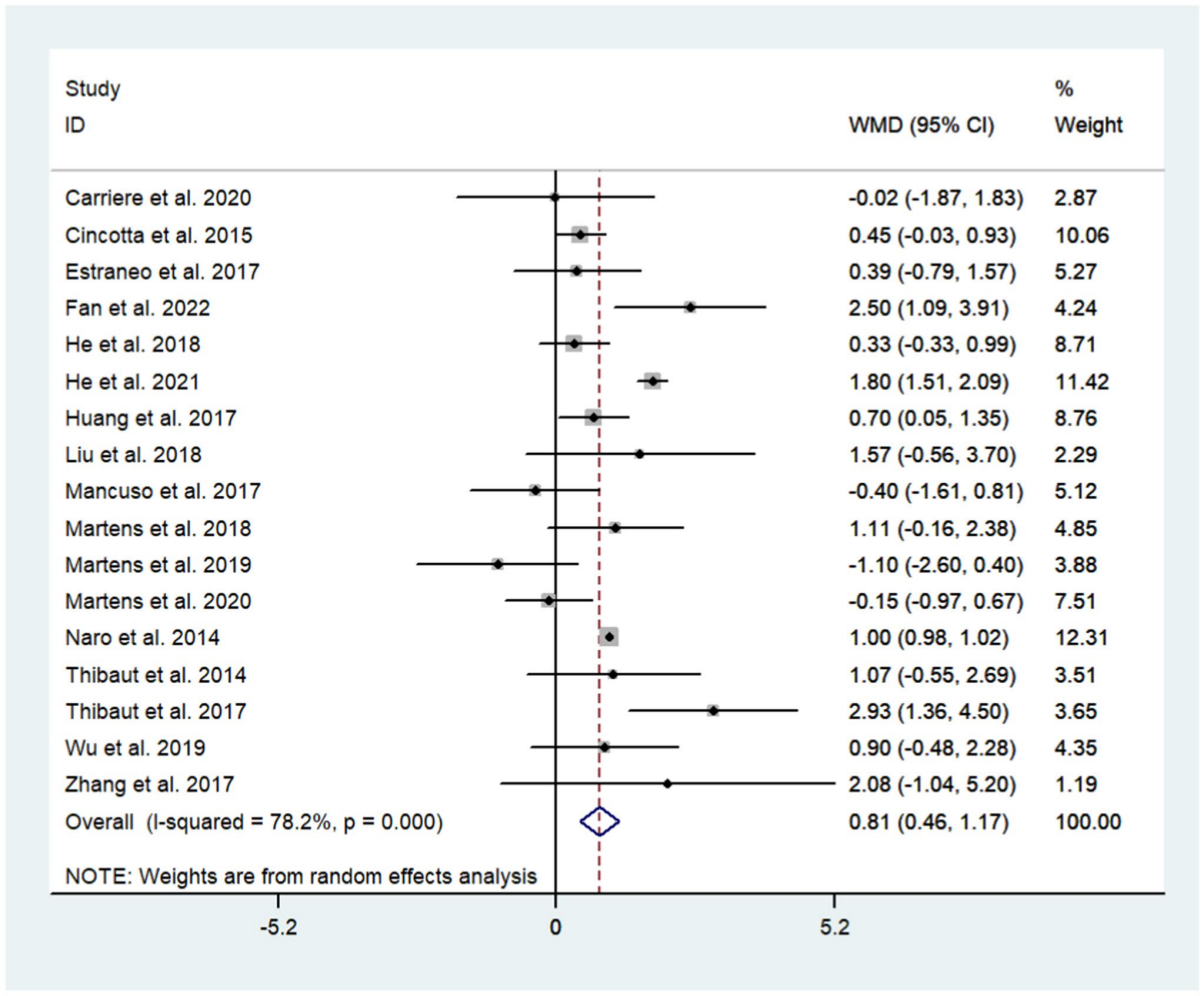
The effects of NIBS on DoC.

**Figure 4:**
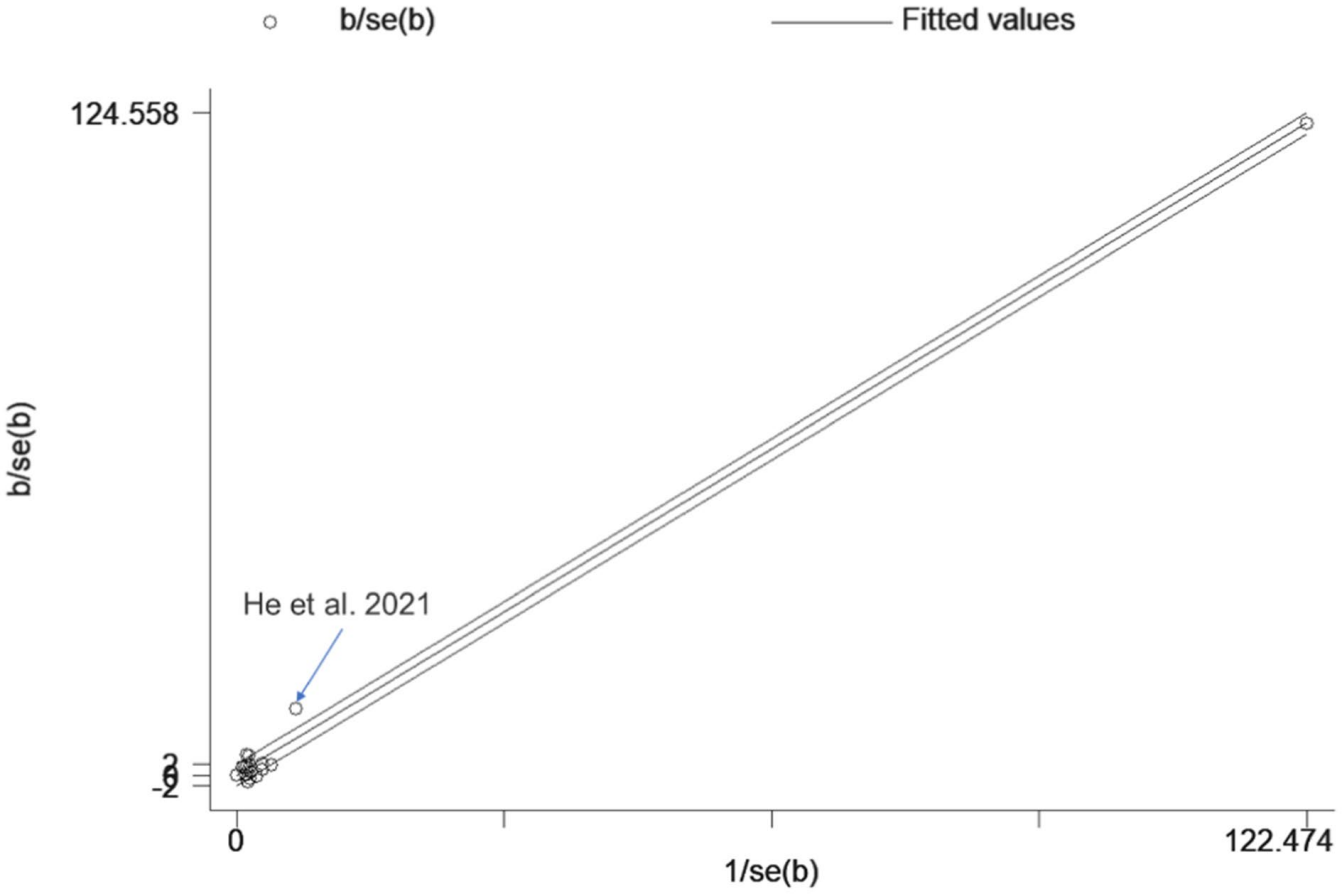
Galbraith plot.

**Figure 5:**
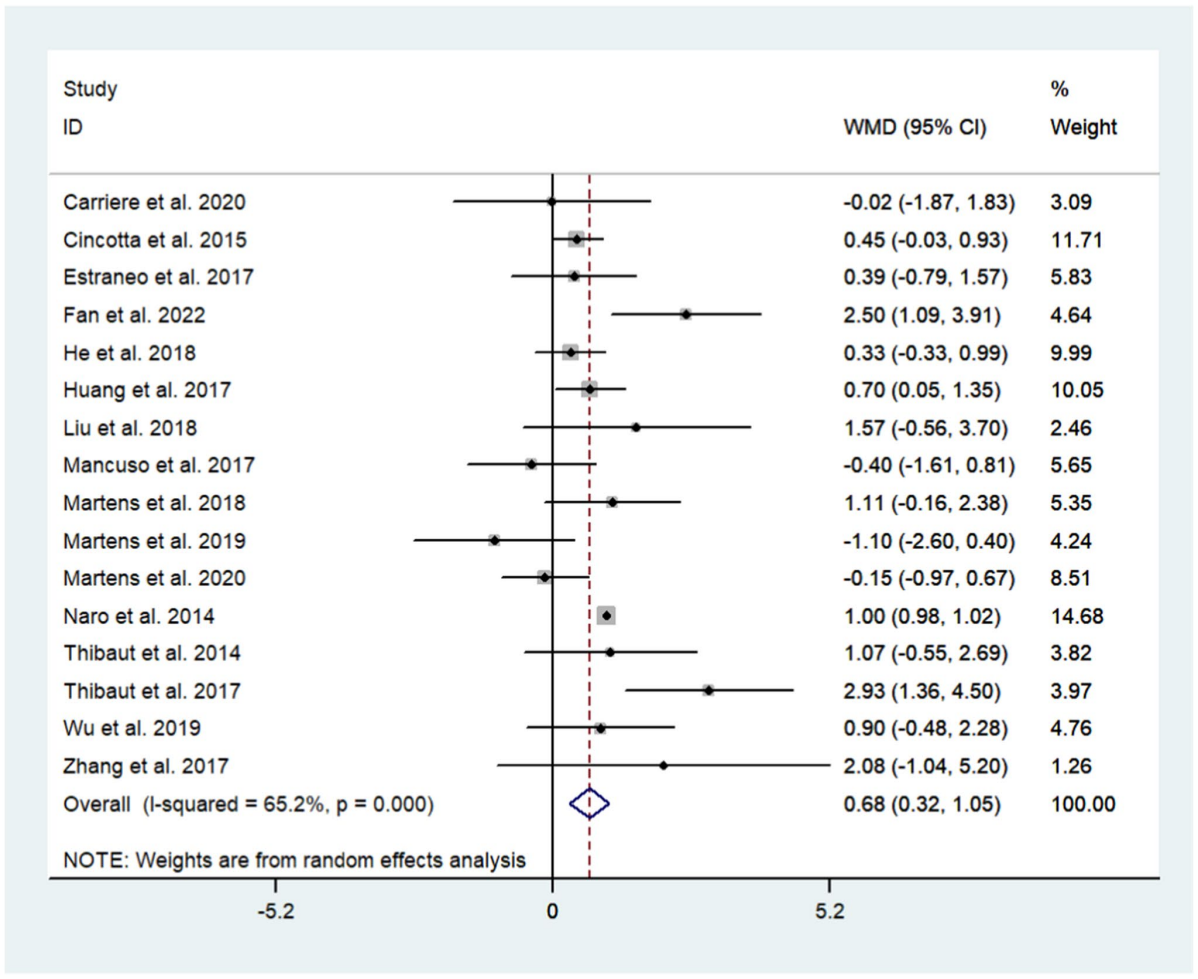
The effects of NIBS on DoC.

A total of 17 studies were included to assess the effects of NIBS on consciousness functioning in patients with DoC. Analysis showed that NIBS treatment significantly improved the con consciousness scious function of patients when compared to the sham stimulation group (WMD: 0.81; 95% CI: 0.46, 1.17; I^2^ = 78.2%, *p* = 0.000, Figure 3). Since I^2^ was >50%, a random effects model was used. The Galbraith plot method was also applied and showed that He et al. (2018) showed large differences when compared to other studies (Figure 4). When excluding this study, there was no significant change in heterogeneity (WMD: 0.68; 95% CI: 0.32, 1.05; I^2^ = 65.2%, *p* = 0.000, Figure 5). When re-examining the study, no clear cause of heterogeneity was found; thus, the study was retained, and the source of heterogeneity was further sought by subgroup analysis and exploring the effects of related factors on clinical outcomes.

### 3.5. Subgroup analyses

#### 3.5.1. Stimulation modality: left DLPFC tDCS vs. left DLPFC rTMS vs. left M1 rTMS

**Figure 6:**
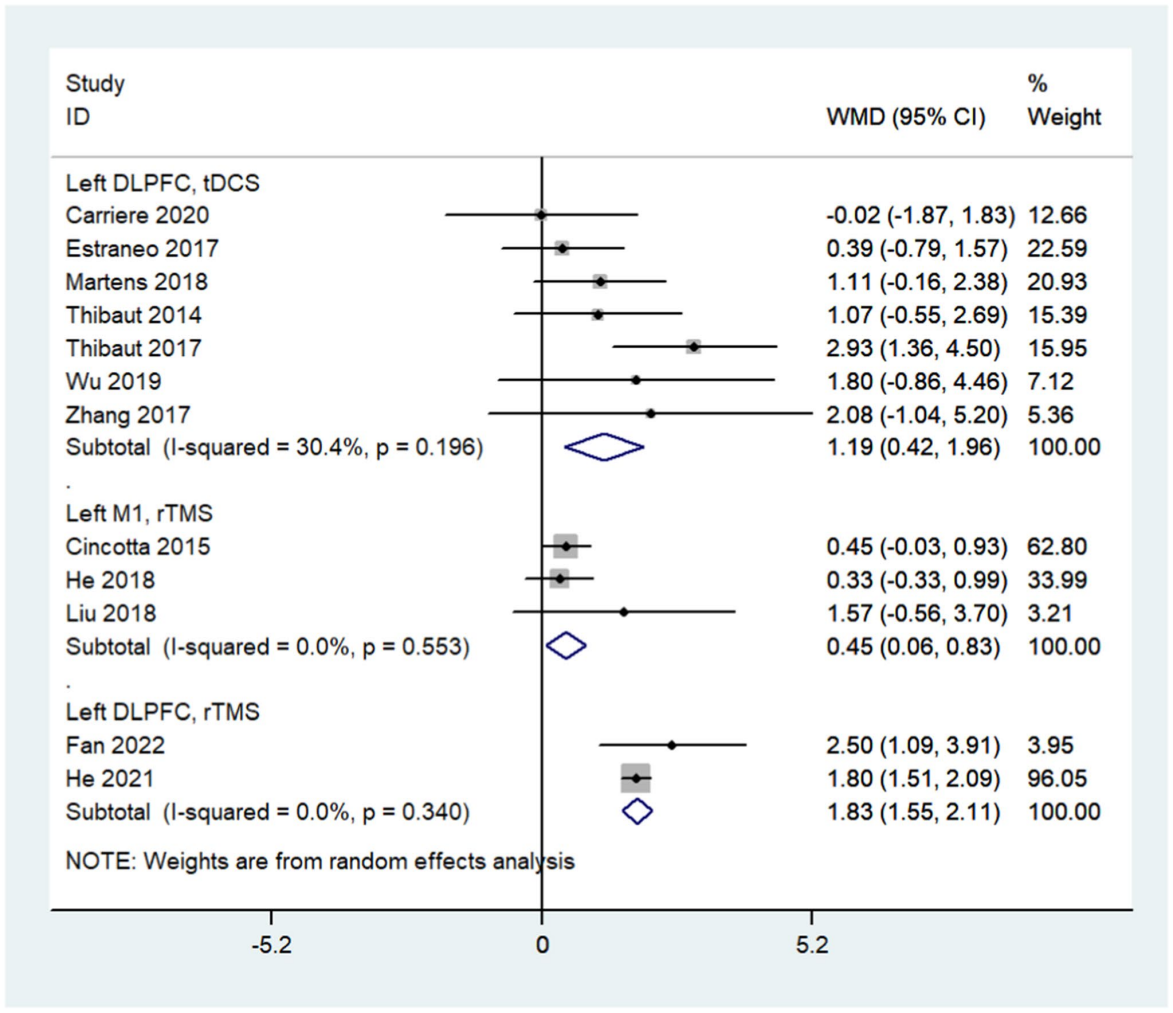
Subgroup analysis based on stimulation modality.

Subgroup analysis based on stimulation modality showed that left DLPFC tDCS (WMD: 1.19; 95% CI: 0.42, 1.96; I^2^ = 30.4%, *p* = 0.196), left M1 rTMS (WMD: 0.45; 95% CI: 0.06, 0.83; I^2^ = 0%, *p* = 0.553) and left DLPFC rTMS (WMD: 1.83; 95% CI: 1.55, 2.11; I^2^ = 0%, *p* = 0.340) all improved the consciousness state of DoC patients (Figure 6). When divided into subgroups by stimulation mode, the heterogeneity of each subgroup was significantly lower than before; different stimulation modes may be the main source of heterogeneity.

#### 3.5.2. Diagnosis: VS/UWS *vs.* MCS

**Figure 7:**
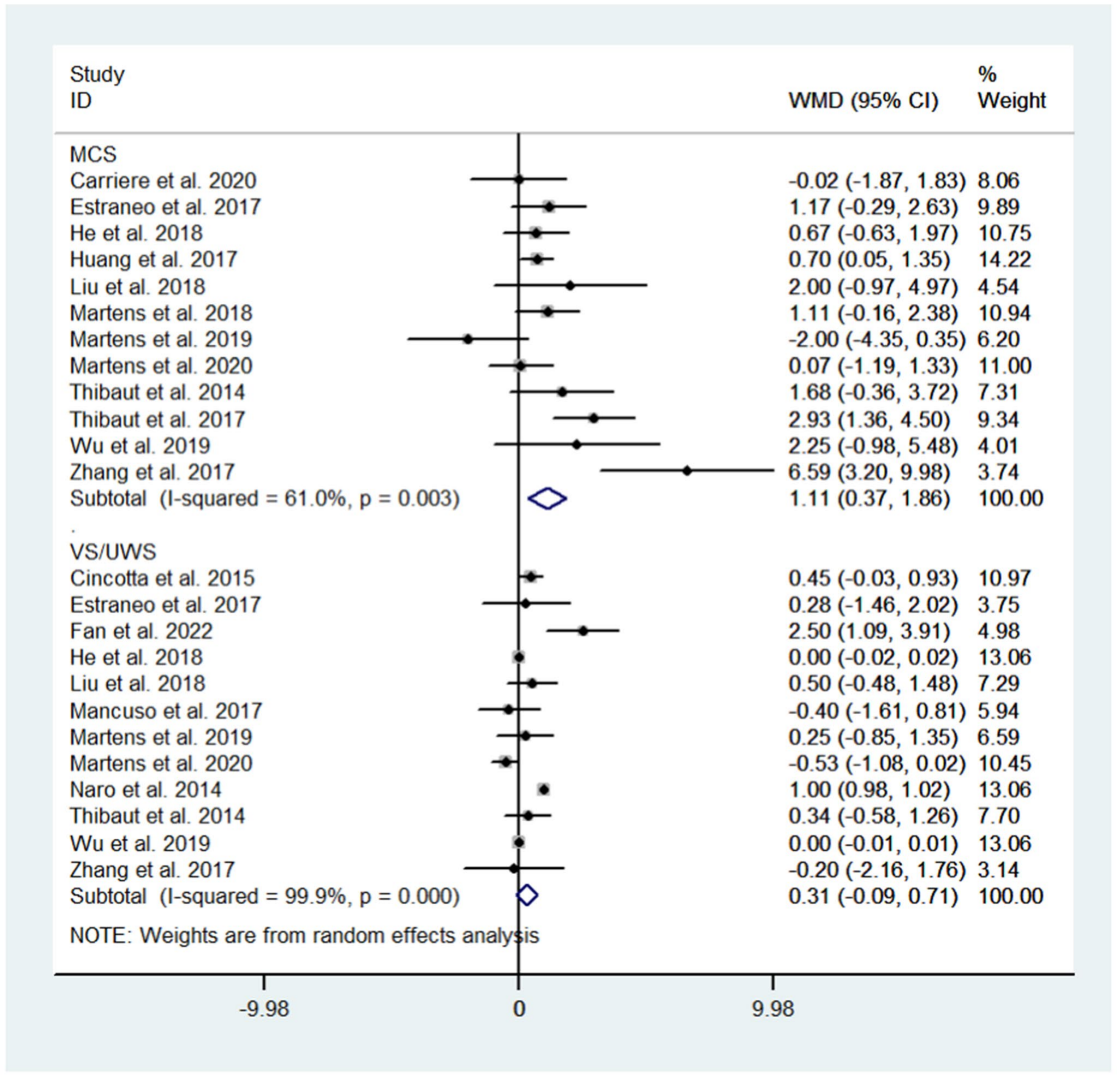
Subgroup analysis based on diagnosis.

Subgroup analysis based on diagnosis showed that NIBS could improve the state of consciousness in MCS patients (WMD: 1.11; 95% CI: 0.37, 1.86; I^2^ = 61.0%, *p* = 0.003) but not in *VS*/UWS patients (WMD: 0.31; 95% CI: −0.09, 0.71; I^2^ = 99.9%, *p* = 0.000, Figure 7).

#### 3.5.3. Observation time: single treatment *vs.* multiple treatments *vs.* long term efficacy

**Figure 8:**
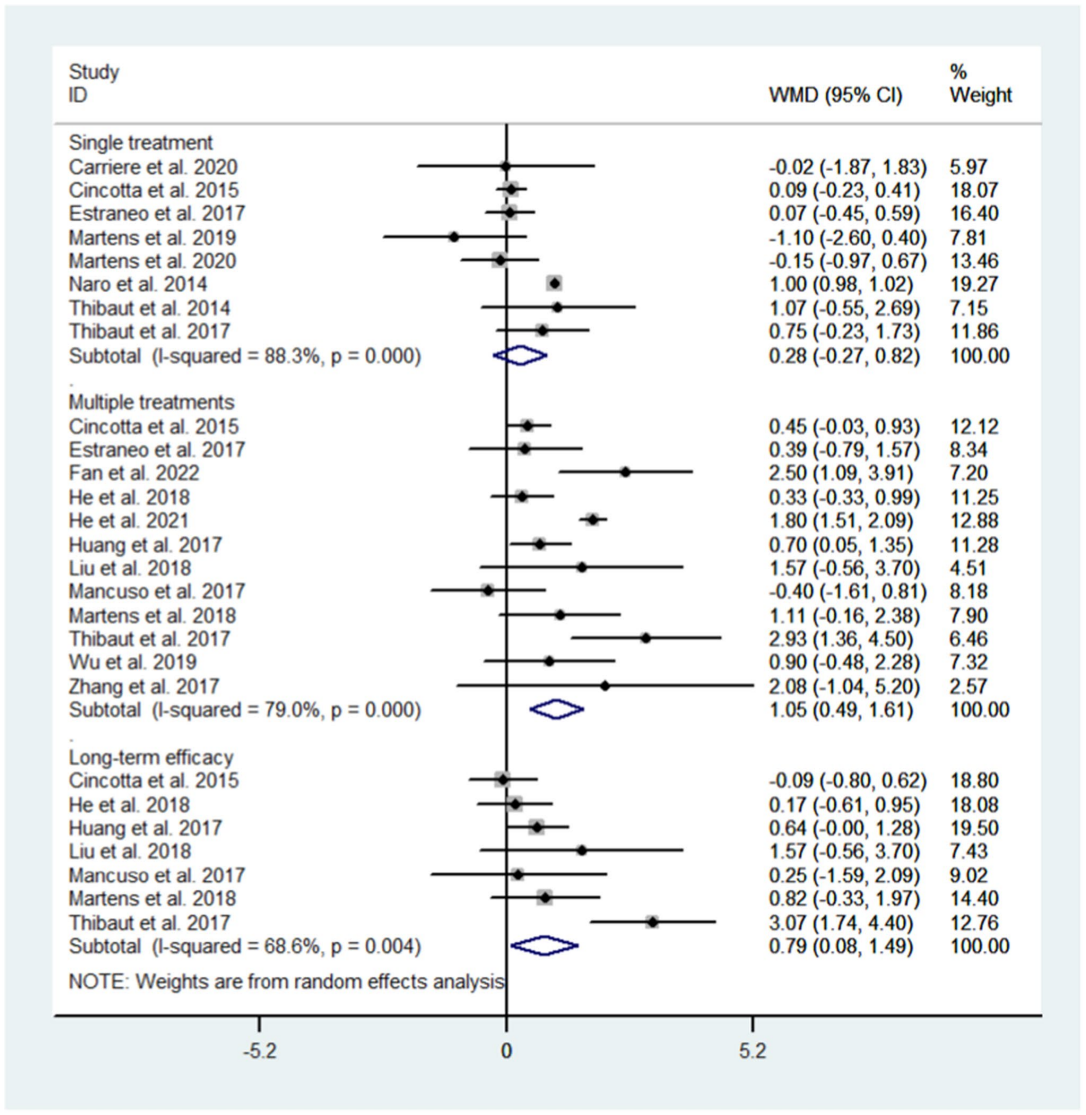
Subgroup analysis based on observation time.

We conducted subgroup analysis for different observation time to evaluate the efficacy of NIBS after single treatment, after multiple treatments, and long term efficacy. The results showed that single treatment did not improve the state of consciousness in DoC patients (WMD: 0.28; 95% CI: −0.27, 0.82; I^2^ = 88.3%, p = 0.000) while multiple treatments did (WMD: 1.05; 95% CI: 0.49, 1.61; I^2^ = 79.0%, p = 0.000); NIBS had long-term effects on DoC patients (WMD: 0.79; 95% CI: 0.08–1.49; I^2^ = 68.6%, *p* = 0.004, Figure 8).

### 3.6. Bayesian analysis

**Figure 9:**
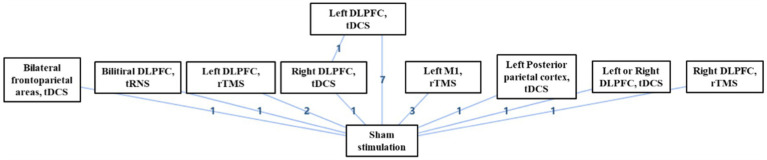
Network diagram of of NIBS on DoC.

**Figure 10:**
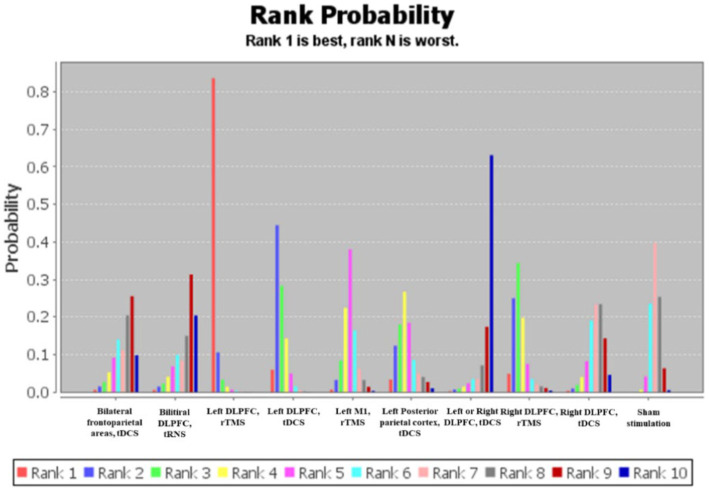
Probability ranking of different interventions.

Of the 17 included studies, 16 studies performed separate comparisons of NIBS with sham stimulation, and one study performed comparisons between left DLPFC tDCS, right DLPFC tDCS, and sham stimulation (Figure 9). Bayesian analysis indicated that left DLPFC rTMS was probably the most significant stimulation modality with regards to treatment effects (Figure 10). The comparative results for various stimulation modalities are detailed in Table 2.

**Table 2:** The meta analysis of network results.

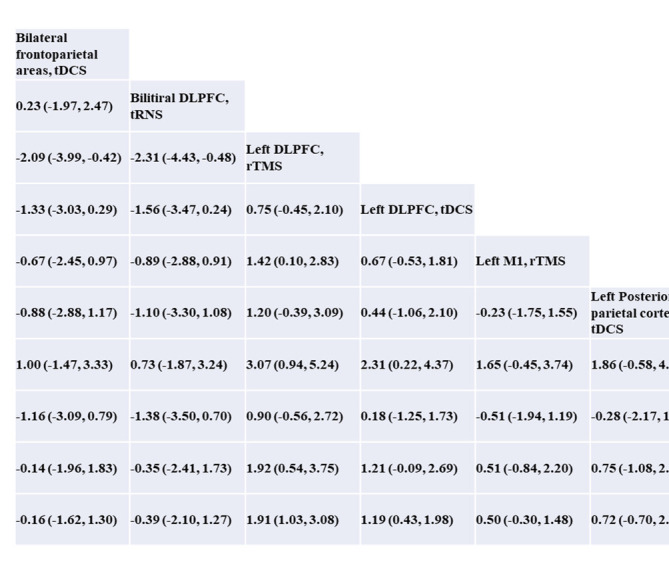

### 3.7. Publication bias and sensitivity analysis

**Figure 11:**
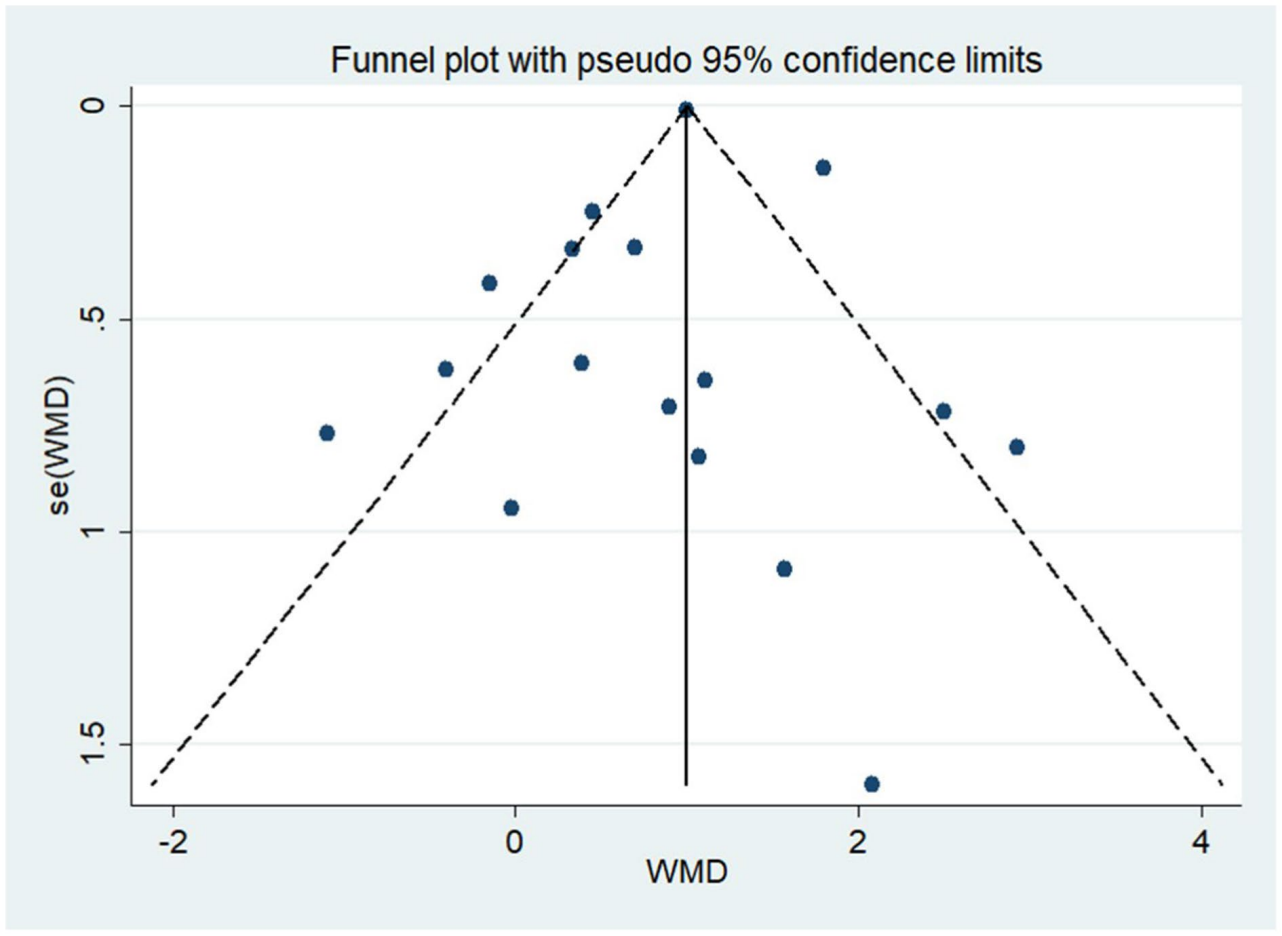
Funnel plot.

**Figure 12:**
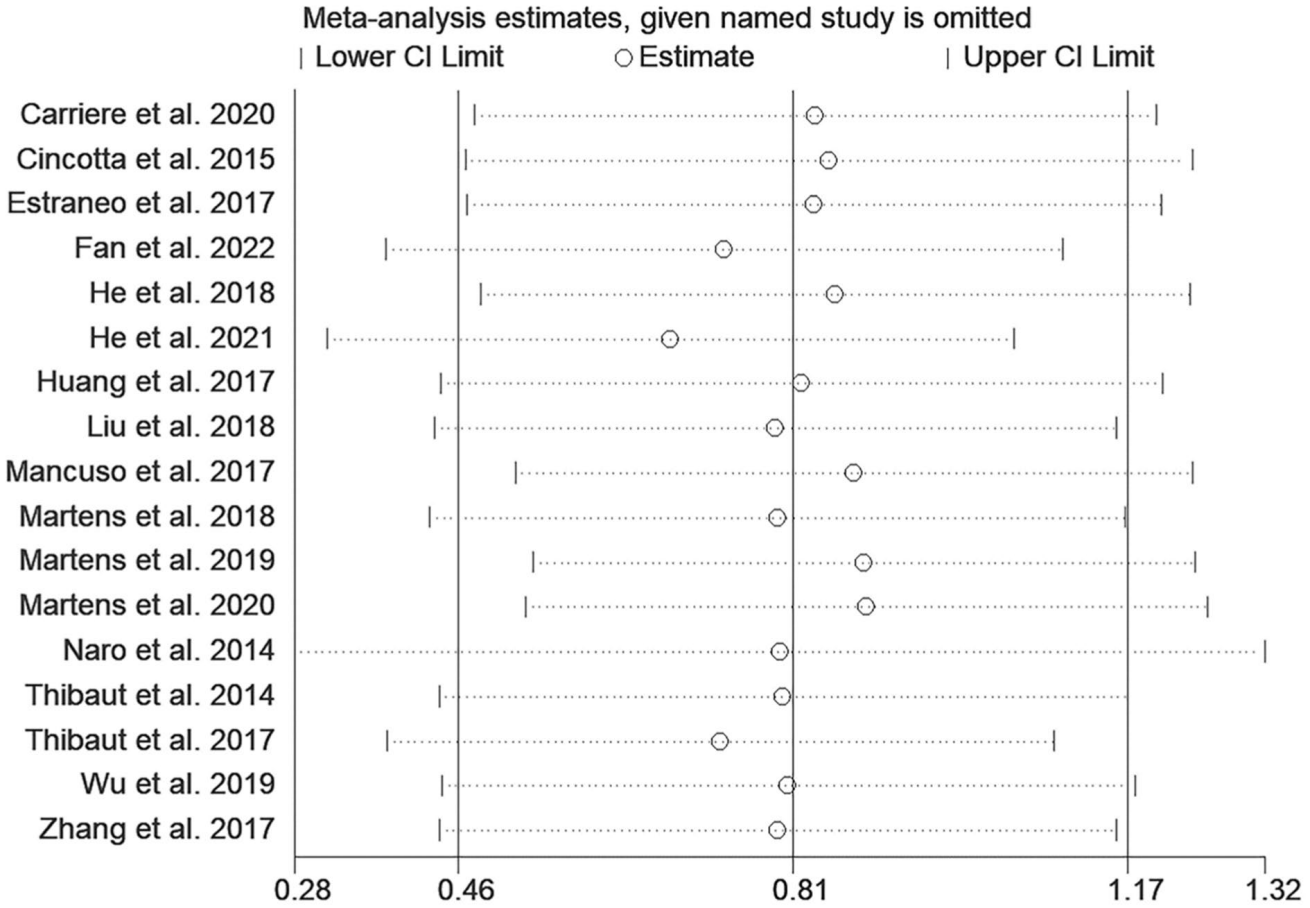
Sensitivity analysis.

As shown in Figure 11, the funnel plot was visually symmetrical, and Egger’s test (*p* = 0.666) further confirmed the absence of significant publication bias. Sensitivity analysis showed that removing any study did not significantly change the effect of NIBS on DoC (Figure 12).

## 4. Discussion

We performed a meta-analysis of patient data from 17 RCTs and included a total of 377 patients with DoC. The overall results show that NIBS improved the state of consciousness in patients with DoC when compared with sham stimulation. Subsequently, we performed subgroup analysis to identify the source of heterogeneity and found that after dividing into different subgroups by stimulation modality, the heterogeneity within each subgroup decreased significantly compared with before, thus implying that stimulation modality may be the main factor affecting the efficacy in DoC patients. On this basis, we performed Bayesian meta-analysis for different stimulation modalities; analysis suggested that left DLPFC rTMS might be the optimal stimulation modality. Furthermore, subgroup analysis based on patient diagnosis suggested that NIBS might improve the state of consciousness in MCS patients but without significant effect in *VS*/UWS patients. We also found that there might be a dose-dependent relationship between the efficacy of NIBS on patients with DoC and identified that multiple treatments could improve a patient’s state of consciousness while single treatments could not. We also found that improvements in a patient’s state of consciousness induced by NIBS were long-standing. Furthermore, our results were free of publication bias and were all stable with regards to sensitivity analyses.

### 4.1. The left DLPFC may be a key target for NIBS treatment in DoC patients

At present, there is no consensus on the targets for transcranial magnetic stimulation in the treatment of DoC; potential targets include the dorsolateral prefrontal lobe, M1 region, frontoparietal lobe, and posterior parietal lobe. Of these, the left dorsolateral prefrontal lobe is the most studied and most recognized target, and was reported in 10 of the 17 included studies (seven tDCS studies, two rTMS studies and one tRNS study) (Thibaut et al., 2014; Estraneo et al., 2017; Mancuso et al., 2017; Thibaut et al., 2017; Zhang et al., 2017; Martens et al., 2018; Wu et al., 2019; Carrière et al., 2020; He et al., 2021; Fan et al., 2022); thus, left DLPFC rTMS is probably the most effective stimulation modality.

The DLPFC belongs to the FPN, an important neural network maintaining consciousness. The main mechanism by which the SLPFC influences the state of consciousness may involve two aspects: on the one hand, the left DLPFC region receives sensory inputs from the parietal cortex including visual, motor, spatial structure and touch, and is closely associated with cognition, emotion and speech function. It has been shown that excitatory stimulation targeting the left DLPFC does not solely affect the stimulation site itself but also improves the excitability of other cortical layers in the frontoparietal network (Sadaghiani et al., 2012; Cavinato et al., 2019). On the other hand, the DLPFC is a critical region of the brain for top-down control and can enhance attention by increasing neural activity in the thalamus and striatum (Miller and Cohen, 2001; Lafontaine et al., 2013); this may facilitate the secretion of important substances that maintain wakefulness, such as dopamine (García-Cabezas et al., 2007; Dandash et al., 2017).

In 2021, He et al. (2018) treated male DoC patients with 20 Hz rTMS over the left DLPFC; a total of 50 patients completed the study. After 10 sessions of treatment, 25 of the patients who underwent active rTMS showed better improvements in CRS-R scores than the sham stimulation group. In addition, patients in the rTMS intervention group had significantly higher serum levels of estradiol after treatment when compared with pre-treatment levels. Changes in estradiol levels were significantly and positively correlated with improvements in CRS-R scores, whereas estradiol levels remained unchanged before and after stimulation in the sham stimulation group. The increase in estradiol levels may set the physiological basis for the successful treatment of DoC patients by rTMS through increased cortical excitability. In 2022, Fan et al. (2022), included 40 patients with DoC and randomized them to 20 Hz active rTMS or sham stimulation over the left DLPFC. After 4 weeks of intervention, patients in the active rTMS group had significantly improved CRS-R scores when compared with those in the sham stimulation group.

In 2018, Martens et al. (2018) performed an intervention on the left DLPFC of MCS patients by tDCS and found that 4 weeks of tDCS led to a modest improvement in the recovery of signs of consciousness in chronic MCS patients. In 2019, Wu et al. (2019) evaluated the efficacy of tDCS therapy in patients with DoC by combining ethology and electroencephalogram (EEG). Fifteen patients with DoC received left DLPFC tDCS, right DLPFC tDCS, and sham stimulation, respectively. After 10 sessions, CRS-R scores improved from pre-treatment in two out of five patients in the left stimulation group and in none of the patients in the right stimulation and sham stimulation groups. EEG results showed that left tDCS increased connectivity between the left and right frontal and parietal cortical areas, and that the internal connectivity of the left frontal lobe was most prominent in four frequency bands. Right-sided tDCS produced enhanced Delta and theta connectivity over a wide range of cortex. No significant effect was observed in the sham group.

In addition, a small number of studies are beginning to apply tRNS in the treatment of DoC patients, although the efficacy of this approach remains uncertain. In 2017, Mancuso et al. (2017) used tRNS targeting the DLPFC in nine *VS*/UWS patients and assessed efficacy by a combination of CRS-R, the Synek scale, an *ad hoc* semi quantitative scale, a clinical global impression improvement scale, and EEG. These authors found no significant difference between the active and sham groups in terms of the improvement of consciousness or the EEG data. Only one patient improved from *VS*/UWS to MCS after tRNS.

In addition to the DLPFC, studies have also focused on the left M1 region. In 2015, Cincotta et al. (2015) conducted a 20 Hz rTMS intervention in the M1 region of *VS*/UWS patients; however, this did not show a treatment effect in terms of behavior and electrophysiology. In 2018 He et al. (2018) used 20 Hz rTMS to intervene in the left M1 region of DoC patients; only one patient with traumatic brain injury showed long-lasting behavioral and neurophysiological changes after 5 days of treatment. The remaining five patients showed brain reactivity at multiple electrodes after undergoing rTMS, although EEG changes were unremarkable. In addition, in 2018, Liu et al. (2018) investigated the use of rTMS in the left M1 region of patients with DoC and observed changes in brain network connectivity before and after stimulation. There were no significant changes in CRS-R scores or FC in patients with DoC after active or sham rTMS stimulation. CRS-R scores were significantly elevated after active rTMS stimulation in one MCS patient with significantly enhanced nodal connectivity in the left lateral parietal cortex (LPC), left inferior temporal cortex (ITC), and right dorsolateral prefrontal cortex (DLPFC).

In 2017, Huang et al. (2017) applied left posterior parietal cortex tDCS on 33 MCS patients. After five days of treatment, CRS-R scores were significantly improved in the active group when compared with the sham group. Regrettably on the fifth day after the end of treatment, there was no significant difference between the CRS-R scores of patients in the active group and those in the sham group. A total of nine (27%) of these patients showed CRS-R improvement during active stimulation, whereas only two patients improved during sham stimulation. Furthermore, in 2020, Martens et al. (2020) used a multifocal tDCS intervention (using 4 anodes and 4 cathodes) over the frontoparietal lobes of DoC patients and showed, at the group level, a significant increase in EEG complexity at low frequencies (1–8 Hz) after tDCS; however, this did not translate into behavioral changes.

### 4.2. MCS patients had a more favorable prognosis after receiving NIBS

Subgroup analysis based on patient diagnosis showed that NIBS could improve the state of consciousness in MCS patients but not in *VS*/UWS patients. The relative *VS*/UWS preservation of function in MCS patients is usually relatively intact and more plastic, and the efficacy of NIBS treatment may depend on the plasticity of the brain.

In 2014, Thibaut et al. (2014) included 30 MCS patients and 25 *VS*/UWS patients to apply anodal and sham tDCS over the left DLPF cortex for 20 min in random order. Clinical evaluation was performed using the CRS-R before and after active and sham tDCS stimulation. Analysis showed that the CRS-R scores of the MCS patients improved when compared to the pre-tDCS state although there was no significant change in the *VS*/UWS patients.

In 2017, Zhang et al. (2017) performed tDCS intervention in 26 patients with DoC, including five *VS*/UWS and eight MCS patients in the active group and six *VS*/UWS and seven MCS patients in the sham stimulation group. After receiving 20 sessions of left DLPFC tDCS over 10 consecutive working days, the MCS patients in the active group improved significantly from their pre-treatment CRS-R scores and showed a significant increase in P300 amplitude. There were no significant changes in behavior or electrophysiology when compared between patients in the active group and the sham group. This suggests that tDCS is more likely to improve the state of consciousness in patients with MCS and that tDCS related improvements in awareness may be related to improvements in the allocation of attentional resources, as reflected by the P300 amplitude.

### 4.3. NIBS probably has a dose-dependent effect on efficacy in DoC patients

Subgroup analysis based on observation time showed that a single treatment did not improve the state of consciousness of DoC patients while multiple treatments (5, 10 or 20 times) could lead to improvements; thus, there might be a dose-dependent and long-term effect of NIBS.

In 2017, Thibaut et al. (2017) targeted MCS patients with left DLPFC tDCS treatment. Analysis showed that a single tDCS treatment did not improve CRS-R scores in MCS patients when compared to sham stimulation, whereas five tDCS treatments did result in improvement. This efficacy persisted 1 week after the end of treatment. In addition, Martens et al. (2018) and Wu et al. (2019) adopted the same intervention to treat MCS patients 20 and 10 times, respectively. After the periodic treatment ended, the state of consciousness improved compared to pre-treatment; however, neither of these two studies reported the effect of single treatment on patients. In 2020, Carrière et al. (2020) showed that a single session of left DLPFC tDCS treatment did not improve the state of consciousness in patients with DoC. Three of the patients showed behavioral improvement after active treatment along with one patient after sham treatment, but this was not significantly different at the group level. It follows that even a single session of tDCS for MCS patients with a relatively good prognosis does not improve the state of consciousness.

### 4.4. Safety of NIBS on DoC patients

NIBS had a high safety profile when applied to DoC patients; none of the included studies reported severe complications in patients when treated with NIBS. Of the 17 included studies, 11 studies clearly reported no adverse effects after NIBS treatment (Cincotta et al., 2015; Naro et al., 2015; Huang et al., 2017; Mancuso et al., 2017; Zhang et al., 2017; Liu et al., 2018; Martens et al., 2019; Wu et al., 2019; Martens et al., 2020), five studies did not indicate adverse effects (Estraneo et al., 2017; He et al., 2018; Carrière et al., 2020; He et al., 2021; Fan et al., 2022), and only one study reported adverse effects (Martens et al., 2018). In 2018, Martens et al. (2018) applied left DLPFC tDCS to MCS patients; 12 patients experienced skin redness (seven during active and five during sham stimulation) and 5 patients reported drowsiness (four during active and one during sham stimulation). One patient who underwent sham stimulation reported seizures and withdrew from the study; it is unclear whether these seizures were related to sham stimulation.

### 4.5. Limitations

There are some limitations in the present study that need to be considered. Although we identified NIBS stimulation modality as a major factor affecting the degree of improvement in CRS-R scores in patients with DoC, partial stimulation modality lacked a sufficiently large sample. Furthermore, we did not consider the impact of etiology on efficacy in DoC patients, as some studies classified TBS and non TBI rather than specific etiologies. In addition, subgroup analysis was only used to identify heterogeneity; thus, the treatment effects in different subgroups should be interpreted with caution. Finally, our search was limited to studies published in English.

## 5. Conclusion

The present meta-analysis suggested that NIBS may improve the state of consciousness in patients with DoC. Subgroup analysis indicated that stimulation modality was the main source of heterogeneity and that left DLPFC rTMS was probably the most effective stimulation modality.

## Data availability statement

The original contributions presented in the study are included in the article/supplementary material, further inquiries can be directed to the corresponding authors.

## Ethics statement

Ethical review and approval was not required for the study on human participants in accordance with the local legislation and institutional requirements. Written informed consent for participation was not required for this study in accordance with the national legislation and the institutional requirements.

## Author contributions

LD and HL performed the literature search, statistical calculations, and drafted the manuscript. HD and XZ performed the bias assessment and participated in experimental design. HZ and SY helped to draft the manuscript. All authors contributed to the article and approved the submitted version.
