# Corrigendum: Efficacy of non-invasive brain stimulation for disorders of consciousness: a systematic review and meta-analysis

**DOI:** 10.3389/fnins.2023.1293703

**Published:** 2023-09-27

**Authors:** Linghui Dong, Hui Li, Hui Dang, Xiaonian Zhang, Shouwei Yue, Hao Zhang

**Affiliations:** ^1^Shandong University, Jinan, Shandong, China; ^2^China Rehabilitation Research Center, Beijing, China; ^3^University of Health and Rehabilitation Sciences, Qingdao, Shandong, China

**Keywords:** non-invasive brain stimulation (NIBS), transcranial direct current stimulation (tDCS), repetitive transcranial magnetic stimulation (rTMS), transcranial random noise stimulation (tRNS), disorders of consciousness (DOC), meta-analysis

In the published article, there was an error in affiliations of Xiaonian Zhang and Hao Zhang. Instead of “Xiaonian Zhang^3^ and Hao Zhang^1,2,3^^*^”, it should be “Xiaonian Zhang^2^ and Hao Zhang^2,1,3^^*^”.

The authors apologize for this error and state that this does not change the scientific conclusions of the article in any way. The original article has been updated.

